# Increasing diversity in genomics requires investment in equitable partnerships and capacity building

**DOI:** 10.1038/s41588-022-01095-y

**Published:** 2022-06-01

**Authors:** Alicia R. Martin, Rocky E. Stroud, Tamrat Abebe, Dickens Akena, Melkam Alemayehu, Lukoye Atwoli, Sinéad B. Chapman, Katelyn Flowers, Bizu Gelaye, Stella Gichuru, Symon M. Kariuki, Sam Kinyanjui, Kristina J. Korte, Nastassja Koen, Karestan C. Koenen, Charles R. J. C. Newton, Ana Maria Olivares, Sam Pollock, Kristianna Post, Ilina Singh, Dan J. Stein, Solomon Teferra, Zukiswa Zingela, Lori B. Chibnik

**Affiliations:** 1 Stanley Center for Psychiatric Research, Broad Institute of Harvard and MIT, Cambridge, MA, USA; 2 Analytic and Translational Genetics Unit, Massachusetts General Hospital, Boston, MA, USA; 3 Program in Medical and Population Genetics, Broad Institute of Harvard and MIT, Cambridge, MA, USA; 4 Department of Epidemiology, Harvard T. H. Chan School of Public Health, Boston, MA, USA; 5 Department of Microbiology, Immunology, and Parasitology, School of Medicine, College of Health Sciences, Addis Ababa University, Addis Ababa, Ethiopia; 6 Department of Psychiatry, School of Medicine, College of Health Sciences, Makerere University, Kampala, Uganda; 7 Department of Psychiatry, School of Medicine, College of Health Sciences, Addis Ababa University, Addis Ababa, Ethiopia; 8 Department of Mental Health, School of Medicine, Moi University College of Health Sciences, Eldoret, Kenya; 9 Brain and Mind Institute, Medical College East Africa, The Aga Khan University, Nairobi, Kenya; 10 Department of Internal Medicine, Medical College East Africa, The Aga Khan University, Nairobi, Kenya; 11 Broad Genomics, Broad Institute of MIT and Harvard, Cambridge, MA, USA; 12 Department of Mental Health, Moi Teaching and Referral Hospital, Eldoret, Kenya; 13 Neurosciences Unit, Clinical Department, KEMRI-Wellcome Trust Research Programme–Coast, Kilifi, Kenya; 14 Department of Psychiatry, University of Oxford, Oxford, UK; 15 Centre for Geographic Medicine Research Coast, KEMRI-Wellcome Trust Research Programme–Coast, Kilifi, Kenya; 16 Nuffield Department of Medicine, Centre for Tropical Medicine and Global Health, University of Oxford, Oxford, UK; 17 Department of Psychiatry, Massachusetts General Hospital, Harvard Medical School, Boston, MA, USA; 18 Department of Psychiatry and Mental Health, University of Cape Town, Cape Town, South Africa; 19 SA MRC Unit on Risk & Resilience in Mental Disorders, University of Cape Town and Neuroscience Institute, Cape Town, South Africa; 20 Department of Psychiatry and Wellcome Centre for Ethics and Humanities, University of Oxford, Oxford, UK; 21 Executive Dean’s Office, Faculty of Health Sciences, Nelson Mandela University, Gqebera, South Africa; 22 Department of Neurology, Massachusetts General Hospital, Harvard Medical School, Boston, MA, USA

## Abstract

Calls for diversity in genomics have motivated new global research collaborations across institutions with highly imbalanced resources. We describe practical lessons we have learned so far from designing multidisciplinary international research and capacity-building programs that prioritize equity in two intertwined programs — the NeuroGAP-Psychosis research study and GINGER training program — spanning institutions in Ethiopia, Kenya, South Africa, Uganda and the united States.

Genetic studies are critical for understanding the molecular etiology of psychiatric disorders, for which diagnostics and therapeutics have lagged considerably behind those in other areas of medicine due to the inaccessibility and complexity of brain tissue^1^. Notably, >80% of participants in genome-wide association studies (GWAS) are of European descent whereas <2% of participants are of African descent, and they contribute disproportionately to discovering novel genetic associations, pinpointing causal variants and aiding polygenic score portability across populations^2–5^. GWAS of psychiatric disorders are no exception, missing significant diversity present on the African continent, where humans originated and retain the most genetic variation worldwide.

The discordance between the widely recognized benefits of diversity in genomic studies versus the dearth of partnerships between high-income and low- and middle-income countries (LMICs) in deploying large-scale genetic studies is indefensible. Furthermore, a legacy of ‘safari research’, in which researchers in high-income countries exploit so-called collaborators in LMICs, has created additional barriers to building successful partnerships. Recognizing the particular benefits of diversity in psychiatric genetic studies for its long-term mission of reducing the global burden of mental illness, the Stanley Center for Psychiatric Research at the Broad Institute of MIT and Harvard in the United States launched the Neuropsychiatric Genetics of African Populations Psychosis (NeuroGAP-Psychosis) project to enroll over 39,000 participants across sites in Ethiopia, Kenya, South Africa and Uganda^6^. Concomitantly, the Global Initiative for Neuropsychiatric Genetics Education in Research (GINGER) launched an immersive training program as part of a collaboration spanning the same institutions and the Harvard T.H. Chan School of Public Health^7^. We discuss here our work in launching the largest genetic study of severe mental illness in African populations to date, our efforts to develop an equitable partnership, the challenges we had to overcome, recommendations for future initiatives aiming to ameliorate Eurocentric study biases based on lessons we learned along the way, and a timeline that describes the realistic level of in-person engagement needed to achieve our goals (Fig. 1).

## Equitable partnerships across resource-imbalanced settings

The NeuroGAP-Psychosis study sprung from a longstanding collaboration between two professors — one in the United States and another in South Africa. As collaborators, they co-mentored a student who later became a faculty member in Kenya, his home country. These three, along with another former student (now in Uganda) and a collaborator from Ethiopia, developed this ambitious project. Strong relationships among these investigators developed over years, providing the critical foundation for the NeuroGAP-Psychosis study. From this tightly knit nucleating group, others were recruited to join the collaboration, showing the power of a few strong partnerships to amplify networks of trust outward. An integral part of this was the early inclusion of experts in ethics — leading to the creation of the Global Initiative in Neuropsychiatric Ethics (NeuroGenE) and the African Ethics Working Group; similarly, the inclusion of experts in pedagogy was critical as it led to the creation of GINGER. Trust has been the foundation for this collaboration, which required continuous reinforcement; without it, corresponding hurdles would have been impossible to navigate.

NeuroGAP-Psychosis is one of the largest genetic studies in each of these institutions and sometimes countries. Consequently, we were constantly told that it would be too hard to accomplish as it forged through uncharted territory. For example, some ethics committees lacked expertise in genetics research and thus had difficulties reviewing our protocols; labs had little experience in carrying out certain required procedures; procuring necessary supplies proved difficult; and each country had different regulations governing the transfer of biological materials. Navigating these hurdles relied on the shared mission of reducing the global burden of mental illness and on the project’s potential benefits (in which GINGER also played an integral role) to all researchers and, it was hoped, in the long term to the communities that contributed as participants. Many project milestones have been met and expectations exceeded, a testament to what can be achieved working together.

Equitable partnerships should be a leading priority for all scientific collaborations; they require initiators to be intentional about building meaningful engagement and equity into every aspect of the partnership by continually evaluating guiding principles and behavior. Continuous feedback loops across all collaborators, regardless of seniority, are critical for solidifying communication, holding candid conversations, expressing feedback and identifying areas for potential growth. Yet success is not guaranteed even with the best-laid plans, necessitating open dialogue and consistent check-ins between partners to ensure that minor setbacks do not turn into large obstacles down the road.

## Lessons and ongoing consequences of inequitable partnerships

Scientists increasingly recognize that safari research is unethical and unsustainable and has a detrimental knock-on effect^8,9^. Trust grows over time but can be shattered in an instant and, once lost, can be difficult to regain. For example, the team in Ethiopia had had negative prior experiences with genetics research whereby data were collected and published with no benefit to local investigators or communities. American and Ethiopian investigators worked together to counter the opposition to genetics research that emanated from this prior experience. This included presenting the study to the Ministry of Science and Technology and the national ethics review committee; as part of this process, investigators responded to questions with a plan for how the study and resulting enhanced research capacity would benefit Ethiopian institutions and participants. This became a reality when one of the laboratories built in this partnership became a COVID-19 testing center.

Many short-term ‘solutions’ to safari research also raise special considerations. For example, donating costly lab equipment quickly ameliorates the resource divide, but without funds to support people to run it, technicians with relevant training, routine maintenance, sufficient bandwidth, dependable electricity and reagents, the equipment is likely to sit unused. Another common solution to resource inequality is to run intensive short courses, namely one- to two-week-long classes on skills associated with the research. Although these are a useful way to bring people together and transfer skills, if the topics taught are not immediately applicable, the courses risk not having a lasting effect^10^. Instead, short courses should be co-designed with local faculty and geared toward local needs and capabilities, and corresponding materials should be designed and transferred with long-term use in mind. Finally, research programs depend on continuity and do not end with data collections or initial publications: funds are required to continue storing samples, analyzing data and translating findings. Whereas grants end in three to five years, capacity building and mentoring is a long-term commitment.

A prevalent indicator of safari research is when data produced by researchers in LMICs are analyzed and published by researchers from primarily high-income countries who have better support to produce these papers quickly. Our work has the greatest impact when more people can use it^11^, and thus we have continually discussed how to ensure that researchers from LMICs can lead publications while also making the data available to outside researchers and other global consortia, resulting in several operational decisions. First, GINGER helps ensure that local scientists in LMICs have skills and mentorship needed to analyze and publish on their own data. Second, our data-sharing policy is based on an H3Africa framework^12^, allowing for a longer embargo period to enable all collaborators to analyze data and publish findings. Finally, when complete, the NeuroGAP-Psychosis data will be included in the Psychiatric Genomics Consortium (PGC), which has invited all NeuroGAP-Psychosis principal investigators and trainees to participate in projects within the PGC.

## Prioritizing education and sharing of ‘know-how’ among partners

Training and mentoring were integrated with research from the outset of our collaboration, primarily through GINGER. This was key to building trust and affirming our commitment to local development beyond sample collections. GINGER leaders visited each NeuroGAP-Psychosis study site to learn about local capabilities and needs from students, faculty and leadership. Then, all NeuroGAP-Psychosis principal investigators traveled to Boston for a two-day ‘curriculum jamboree’ to lay foundations for the GINGER program along with additional experts in research, pedagogy and ethics. GINGER benefited from insights shared by other virtual and hybrid training programs and, in turn, Harvard colleagues learned about practical barriers for researchers from LMICs and requirements for a successful research career in their countries.

Ultimately, the goal of GINGER is to support a cadre of scientists to locally lead African mental health genetics and contribute scientifically to the global research community^13^. Building on lessons from the curriculum jamboree, the GINGER program rests on three pillars of support: support from above via assigned mentors, support from within via institutional involvement and the GINGER program team, and support to the next generation via institutional training and building mentoring skills. GINGER consists of three programmatic components: (i) a Research Fellows program (Table 1), (ii) an Institutional Training program and (iii) a Teaching Fellows program.

The **GINGER Research Fellows program** provides tuition-free, multi-year training for early-career researchers at NeuroGAP-Psychosis collaborating institutions to enable each fellow to lead neuropsychiatric genetics analysis in their countries. Fellows participate in a combination of in-person and virtual learning. The short, in-person workshops teach topics that are difficult to teach in a virtual environment, formulating group projects, building professional skills including time management and science communication^13^ and providing the foundation for establishing close connections between fellows, building intra-African networks and research collaborations. Weekly live and pre-recorded virtual classrooms maintain connections built during the in-person workshops while locally enabling access to top-tier instructors from around the globe (especially important for fellows with children). GINGER’s expertise in virtual learning became unexpectedly helpful for all participating institutions after the COVID-19 pandemic halted in-person events globally.

The **Institutional Training program** trains undergraduate and graduate students from the broader communities at collaborating institutions. Each institution collaboratively identifies topics and co-develops teaching materials with GINGER faculty, enabling them to be embedded in the institutional curriculum via existing courses or as independent short courses. GINGER optimizes reusability of materials and encourages the inclusion of local faculty to serve as teaching assistants in the classes, so that new topics are covered in future training. The **GINGER Teaching Fellows program** invites early-career researchers from the Stanley Center and Harvard-Chan to develop curriculum and teach, leading to some collaborations between GINGER Research Fellows and Teaching Fellows that have led to papers^14^ and successful grant applications.

Designing GINGER came with unique challenges. Some were expected and planned for. For example, we purchased hardware enabling stronger internet access and required all training videos to be downloadable to reduce streaming. Still, lessons on cloud computing or requiring virtual private network (VPN) access were especially challenging, as even the strongest bandwidth available is not always sufficient for all requirements. Additionally, sometimes our fellows experience power rationing or suspension of the internet. When this happens, we are flexible and work with our fellows to make sure they can stay on track. Many fellows have very limited access to journals at their home institutions, so a small yet highly impactful decision was fighting for them to have access to the Harvard Medical School Countway Library.

## Continuously addressing gaps in infrastructure and skills

We started NeuroGAP-Psychosis with the acknowledgment that resources and infrastructure are inherently imbalanced between researchers from high-income countries and LMICs. Our group dedicated time, resources, education and training to counteract imbalances in the limited ways that we could. From the outset, we agreed that success was to be measured not only by the data collected, papers published or scientific discoveries; discussions were equally focused on sustainability, capacity building and infrastructure. This required funding flexibility and has led to earlier, unanticipated and wider-reaching benefits beyond our research goals.

### Wet-lab capacity

Research partners across the five recruiting institutions each had very different wet-lab capacities when protocols were being developed. A major aim was to expand research capacity to support each wet lab to do most work locally, including sample collection, extraction and storage. This goal has been met, with only small aliquots of DNA needing to be sent to the Broad Institute, the only participating institution with facilities currently capable of sequencing tens of thousands of genomes. Members from the Broad Institute’s Genomics Platform traveled to study sites to meet with laboratory staff, review research protocols, streamline organizational methods, assist in developing standard operating procedures and ensure that best practices were being followed in the lab. The goal was not to make each lab equal, but to candidly determine what the project could do to facilitate success by helping each lab advance.

For Moi Teaching and Referral Hospital in Eldoret, Kenya, that meant providing training on how to use a NanoDrop spectrophotometer, funding MS and PhD student research and discussing sample tracking (i.e., chain of custody) options. Sam Pollock from Broad’s Genomics Platform visited multiple collaborating wet labs and described sample tracking as an area of focus in the absence of a robust laboratory information management system (LIMS). After he had shadowed lab members, they discussed and settled on best practices including using printed Excel tracking sheets, numbering Eppendorf tubes and assigning responsibility when recording sample accession.

For Addis Ababa University (AAU) in Addis Ababa, Ethiopia, that meant streamlining lab work flows and assisting with procurement hurdles. Creating freezer maps provided an organizational tool for use across multiple groups, which simplified communication and reduced the risk of samples breaking the cold chain. Although AAU did not have a LIMS, risk of sample swaps was mitigated through visual management, sample batching and use of Excel formulas as verification tools. NeuroGAP-Psychosis funding helped establish the first biobank in Ethiopia at the College of Health Sciences at AAU through lab support and funding for freezers. An unanticipated benefit was also the support it provided to PCR test patients for SARS-CoV-2.

### Annual general meetings

Before the pandemic, we held in-person annual general meetings (AGMs). Unlike in many other scientific consortia that involve only principal investigators and a few analysts in these meetings, key project personnel spanning all career stages, including principal investigators, research assistants, Scientific Advisory Board members, GINGER fellows, wet lab staff and project managers, attended the meetings and engaged in critical discussions. The AGMs were designed to advance research, provide training and enable discussion of strategies and goals for the next year. Beyond this, they established and built persistent relationships among a network of 100+ researchers, clinicians, ethicists and staff across all sites. These unique benefits made them well worth the many logistical and administrative hurdles, hefty financial cost, time and effort involved. To quote a principal investigator and author, Dickens Akena of Makerere University in Uganda, summarizing the value of the AGMs: “There is something about meeting with people you work with that is so indescribable—something you look forward to. There is always something new, something to learn, an opportunity to get better and to improve careers. The unpredictability is constant.”

The AGMs propel NeuroGAP-Psychosis forward by creating an atmosphere conducive to sharing ideas on a more personal level. Discussions involved sharing experiences between teams, identifying and surmounting challenges, preliminary data analysis, data generation strategies^15^, mentoring GINGER fellows and showcasing their work, clinical training needs surrounding patient interactions, funding opportunities and ethical challenges^16^. They also built camaraderie from shared experiences not possible over conference calls. Most importantly, AGMs gave junior researchers opportunities to meet and partner with senior researchers from other institutions and consortia. The AGM itself typically occupies only 1-2 days, but there are also many adjacent meetings and retreats, including GINGER training sessions, African Ethics Working Group meetings and project manager retreats.

### Team building and engagement via project manager retreats

Project managers arrived in the host country a few days before the AGM to see firsthand how other clinics worked and improve their own workflows locally; they also met and discussed all areas of research operations, including recruitment strategies, sample tracking and shipping logistics. Project manager training sessions were often led by project managers themselves in areas in which they were subject matter experts, with the US team only facilitating. Project managers learned how other clinics and teams at different sites operated and had candid discussions on, for example, how best to approach patient interactions, assess inter-rater reliability, interpret data trends, ensure the highest data quality, supervise and mentor their research assistants, and what to do when the technology inevitably breaks. These training sessions provided opportunities to standardize administering phenotyping tools, adhering to updated research protocols and overcoming common challenges across all sites. Talking through successes and challenges faced by all project managers fostered lasting relationships.

**Fig. 2:**
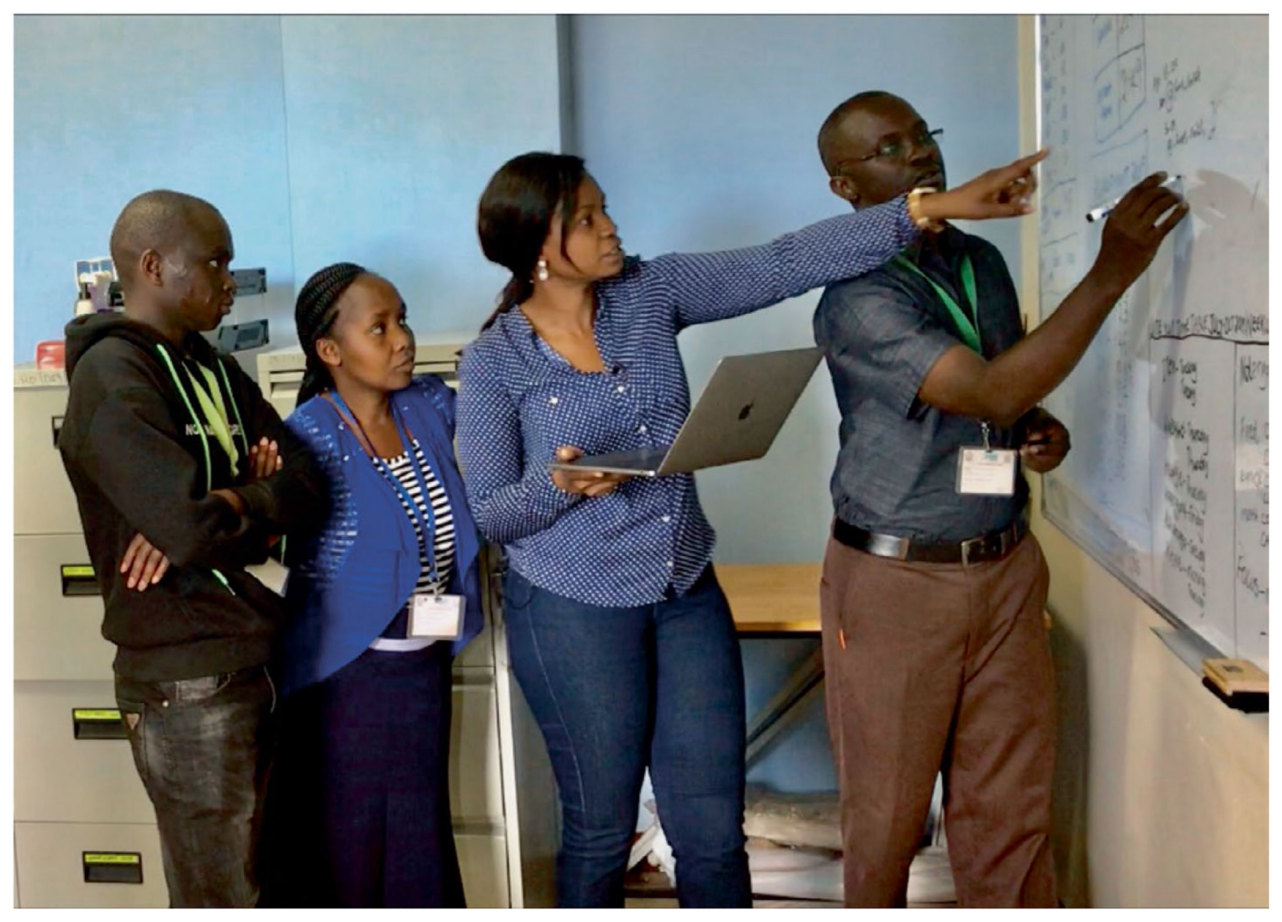
Project manager Stella Gichuru discusses NeuroGAP-Psychosis with research assistants. From left to right: Fredrick Ochieng, Eunice Menjo, Stella Gichuru and Wilberforce Ndenga. Photo credit: Russell Murachver.

Project manager and author Stella Gichuru at Moi University describes how this cohesion aided the project practically: “Whenever one project manager faced a challenge, there was great team spirit and cooperation in resolving that matter as a project manager team. We would come together, either in the annual project manager meetings or through our WhatsApp group, to ensure uniformity in operations across all sites.” For example, she described training in using the Mini International Neuropsychiatric Instrument (MINI) for conducting structured diagnostic interviews as a major focus of the project manager retreat in 2018. Beforehand, each project manager had their own understanding of the MINI, but through discussions and role plays, they left with a uniform understanding that they then passed to research assistants (Fig. 2). Melkam Kebede, the project manager from AAU and a GINGER fellow and author, felt that these project manager retreats allowed participants to see the ‘bigger picture’, outside the typical day-to-day operations of running a data collection team in their own country, and provided the chance to hear and work through problems that other sites were facing.

## Challenges with international partnerships persist

The NeuroGAP-Psychosis and GINGER programs were generously supported philanthropically via the Stanley Center. Although we describe these programs as positive examples of collaborations across resource-imbalanced and multidisciplinary settings, we recognize our privilege and funding flexibility, acknowledge that we still fell short in several areas and describe some persistent challenges^17–19^.

Traditional funding mechanisms for projects like NeuroGAP-Psychosis are most abundant in high-income countries, with contact principal investigators typically having higher success rates obtaining funding when they live in those countries. Funders often provide low overhead for overseas institutions, do not provide resources for training on grant management and yet require LMIC institutions to meet their reporting standards. Additionally, because of this, most decisions are made by scientific leaders from high-income countries, reinforcing hierarchies rather than forming long-term partnerships among LMICs. These imbalances affect regional networking and sustainability.

Brain drain—the emigration of highly skilled researchers—impacts a country’s development potential and an institution’s reputational capital. Prioritizing capacity building in LMICs is critical for sustaining equitable partnerships. Yet training programs like GINGER when offered by foreign collaborating institutions often have unclear accreditation, and although samples remain local to each site in NeuroGAP-Psychosis, it is not yet clear who will lead future research programs with these samples. Ensuring that such programs actually help trainees move their careers forward requires careful consideration of the local reward system.

More proximally than these structural issues, NeuroGAP-Psychosis missed some opportunities that could have aided this and downstream projects even more. For example, at some project sites, NanoDrop instruments used for DNA quantification are not being used outside the NeuroGAP-Psychosis project, suggesting that other support might have had more local value. Sam Pollock says, “I think expanding and updating the LIMS for the labs would’ve been the most impactful opportunity that we weren't able to do. The cost and time needed was too much and there were other small improvements that needed to be completed during the limited time we had.” Furthermore, the most active feedback loops were horizontal rather than across hierarchies. The most critical communication usually happened in person, initially precipitated by tough conversations about what needed to take place and resulting requirements. NeuroGAP-Psychosis and GINGER could be further enriched by more consultation with other longstanding training programs that exist among some of our collaborating institutions^20^.

## Recommendations for building equitable research partnerships

We share five takeaways from our partnerships across resource-imbalanced settings. First, local collaborators are essential to the success of the project and future work. NeuroGAP-Psychosis principal investigators have longstanding relationships that were critical for establishing a network of connected researchers across sites. Second, clear and detailed collaboration agreements are needed at the outset of the project that lay out explicit rules of engagement, including details such as the process for accessing data, data-sharing policy, embargo policies and authorship policy. NeuroGAP-Psychosis benefited from existing policies developed by H3Africa and the Psychiatric Genomics Consortium. Third, funding needs to be flexible, adapting to site-specific project needs that can be difficult to anticipate at the outset, such as sample transport, storage and internet connectivity costs. For NeuroGAP-Psychosis, philanthropic funding covered these costs even though they were not originally budgeted for, whereas NIH funding would require them to be budgeted before grants were awarded. In addition, funds may be needed up front rather than via reimbursement. Fourth, in-person meetings are critical for frank and honest feedback. NeuroGAP-Psychosis AGMs advanced trust, knockoff studies, communication of real-time challenges and consistent implementation of protocols^17,21^. In-person GINGER training was essential for solidifying concepts, debugging computational setups, developing hands-on skills difficult to teach remotely, building trust among fellows and advancing projects led by the GINGER fellows^14^. Fifth, research missions are best advanced when commensurate attention is given to capacity building. NeuroGAP-Psychosis would have been impossible without GINGER because GINGER helped build trust and sustainability. Beyond NeuroGAP-Psychosis and GINGER, the ultimate goal of equitable science requires systemically prioritizing diversity. Key pillars for building and sustaining such efforts include stakeholder will, infrastructure, strategic funding, capacity building and global partnerships with consortia^22^.

## Figures and Tables

**Fig. 1:**
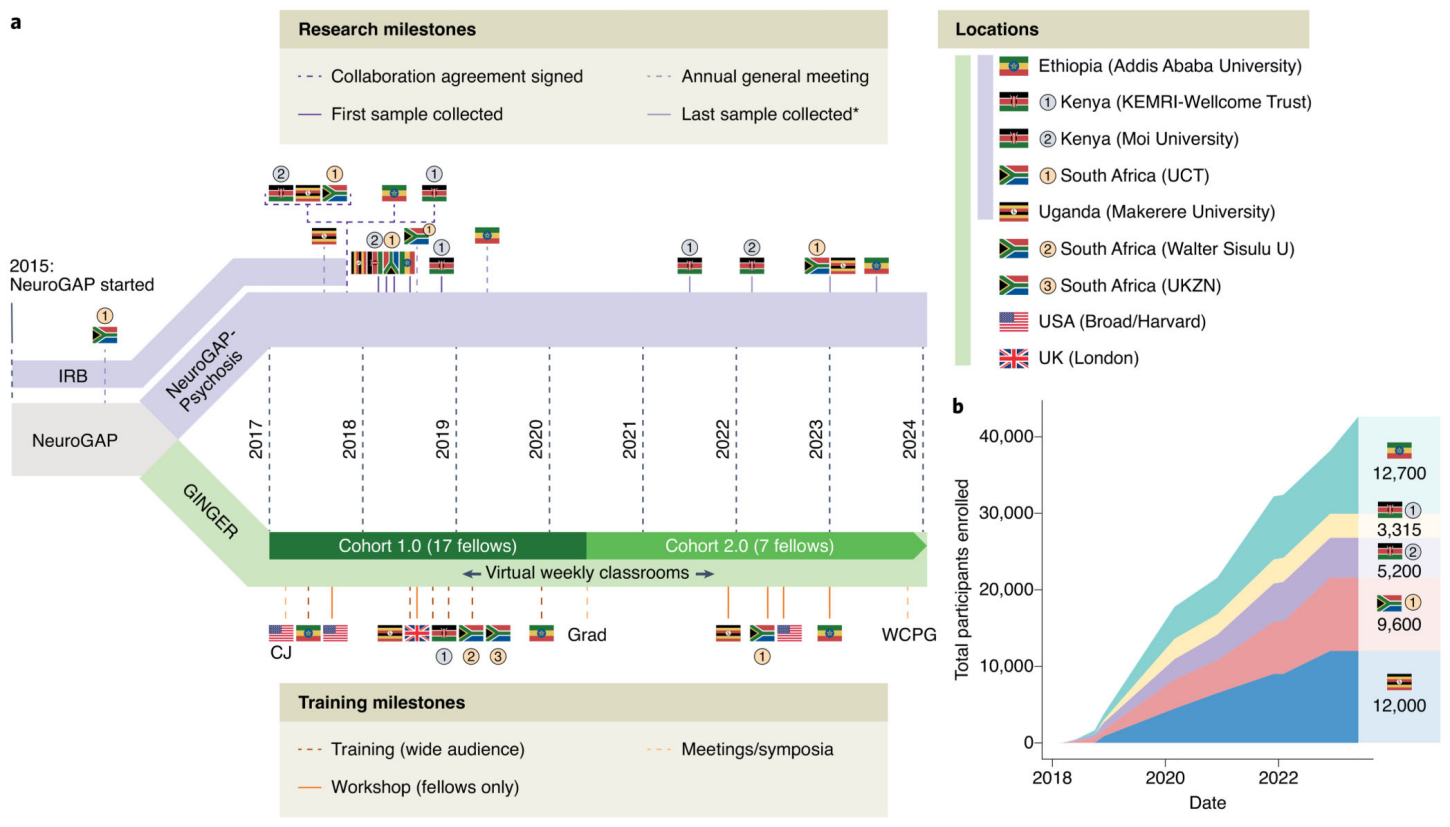
Timeline of NeuroGAP-Psychosis and GINGER Program to date. **a**, Overview of NeuroGAP Project milestones relating to research in NeuroGAP-Psychosis and capacity building in GINGER. CJ, curriculum jamboree; Grad, virtual graduation ceremony for GINGER Cohort 1.0; IRB, Institutional Review Board; UCT, university of Cape Town; UKZN, university of KwaZulu-Natal; WCPG, World Congress of Psychiatric Genetics. *For ongoing studies, some dates for last sample collection are in the future. Research protocols were initiated in 2015 (indicated by IRB). Collaboration agreements were signed by each institution following approval by respective local ethics review committees and IRB approval. **b**, Enrollment of participants in NeuroGAP-Psychosis over time.

**Table 1:** Summary of productivity metrics from the first GINGER cohort (2017–2020)

GINGER program metric description	Count
GINGER Research Fellows	17
People who attended the onsite trainings (approximate)	230
Local teaching assistants	6
Guest speakers in the virtual classrooms and workshops	52
Hours of curriculum from the virtual classrooms	126
Hours of curriculum from on-site trainings	262
PhDs started/completed	3 / 3
Promotions	8
Fellowships earned	3
Publications (unique)	70
Group publications	2
First publications	6
Teaching Fellows (overall)	24
